# The Impact of a Dissonance-Based Eating Disorders Intervention on Implicit Attitudes to Thinness in Women of Diverse Sexual Orientations

**DOI:** 10.3389/fpsyg.2019.02611

**Published:** 2019-11-29

**Authors:** R. M. Naina Kant, Agnes Wong-Chung, Elizabeth H. Evans, Elaine C. Stanton, Lynda G. Boothroyd

**Affiliations:** ^1^Department of Psychology, Durham University, Durham, United Kingdom; ^2^School of Psychology, Newcastle University, Newcastle upon Tyne, United Kingdom

**Keywords:** cognitive dissonance, body image, intervention, eating disorders, implicit attitudes

## Abstract

Dissonance-based body image programs have shown long-term effectiveness in preventing eating disorders and reducing risk factors for eating disorders in women. Here we report on the potential for one such intervention to impact on implicit attitudes toward thinness as well as an explicit measure of eating attitudes, across a sexually diverse group of young women. The Succeed Body Image Programme was adapted to remove heteronormative assumptions and was delivered to a final sample of 56 undergraduate women who reported their sexual orientation as either “predominantly heterosexual” (our term; 1 or 2 on a 7-point Kinsey scale, *n* = 38) or non-heterosexual (3–7 on the Kinsey scale, *n* = 18). Before and after the intervention, they completed the Eating Attitudes Test-26, and an associative reaction time task based on the Implicit Association Test, in which bodies of low and higher weight were paired with socially desirable or undesirable traits. A total of 37 predominantly heterosexual women completed a control intervention in which they read NHS leaflets on eating disorders and healthy weight. Results showed that the intervention made predominantly heterosexual participants less prone, versus control, to associating thinness with positive traits on the IAT and all women completing the intervention reported a lower level of disordered eating attitudes at post- than pre-test. Non-heterosexual women, however, showed a non-significant increase in thin-bias on the IAT, perhaps due to their low baseline. These results imply that intensive dissonance-based programs can change attitudes at the automatic, implicit level as well as merely giving women tools to overcome those implicit attitudes.

## Introduction

Eating disorders are not only accompanied by a wide range of chronic symptoms functionally impairing the whole body but also increase the future risk of depression, obesity, and anxiety disorders among other psychological issues (Stice and Bearman, 2001; Stice et al., 2013). They have the highest mortality rate of any group of psychiatric disorders (Harris and Barraclough, 1998; Keshaviah et al., 2014), and relapse rates following treatment typically exceed 30% (Olmsted et al., 2015; Berends et al., 2018). Treatment attrition is similarly high (DeJong et al., 2012; Vall and Wade, 2015), underscoring the importance of efforts to prevent the initial development of disordered eating symptoms in higher risk populations, such as young women (Austin, 2016).

Key risk factors in the development of eating disorders are negative body image and appearance pressures (e.g., Stice, 2002; Stice et al., 2010), in particular a focus on achieving an unrealistically proportioned, unattainable thin ideal commonly promoted in Western media (Tiggemann and Miller, 2010; Cash and Smolak, 2011). Efforts to reduce eating disorder prevalence have found some success in the use of dissonance-based persuasion theories (Stice and Shaw, 2004; Becker and Stice, 2011). Indeed, a recent review concluded that dissonance-based prevention interventions were most effective in reducing eating disorder risk for higher risk populations (Watson et al., 2016). One such intervention is the Succeed Body Image Programme (SBIP; Becker and Stice, 2011, 2012), a UK adaption of peer-led workshop, Reflections, originally delivered *via* American sororities (Becker et al., 2005). A key aspect of the program requires women to actively criticize the thin-ideal standard of beauty *via* roleplays, letter writing, group discussions, and self-affirmation exercises. It is proposed that the inconsistency between these behaviors and their previous internal attitudes generates cognitive dissonance, which participants resolve by decreasing their internal adherence to the thin ideal, resulting in decreased body dissatisfaction, and decreases in eating disorder symptoms and unhealthy weight control behavior long after the intervention is finished (Becker et al., 2005). Indeed, this approach shows long-lasting improvement in eating disorder risk factors and symptoms in various environments for at least 3 years (e.g., Stice et al., 2008; Kilpela et al., 2014).

The current study sought to further understand the effectiveness of an eating disorder prevention program on two counts. Firstly, our primary aim was to assess the implicit versus explicit change in participants’ attitudes following the intervention in order to better understand the degree to which the program is truly internalized. We also had a secondary aim to consider how broadly applicable the program was to women of diverse sexuality.

### Implicit Attitudes

Despite well-documented decreases in eating disorder risk, however, the mechanism by which these improvements come about remains opaque. While cognitive dissonance purports to induce genuine change in attitudes, the outcomes tested in the research into eating disorder prevention have almost entirely been explicit, self-report measures which are inherently open to self-report bias (for key reviews see, e.g., Stice and Shaw, 2004; Pennesi and Wade, 2016; Stice et al., 2019). For instance, it may be that participation in body-positive interventions helps participants to ignore their internalized thin ideals without actually changing those internal ideas, or the intervention may lead them to believe that reporting such ideals is socially undesirable, and thus under-report their internal ideals and eating disorder symptomology.

One study of note avoided the risks of report or presentation bias by considering fMRI evidence, finding that participants who completed the dissonance program showed reduced responsivity in reward pathways, specifically in the caudate, when viewing stimuli representing thin models (Stice et al., 2015). The current study investigated the impacts of dissonance programs on internal attitudes through another means: by testing whether participation in the Succeed program can alter not only traditional explicit measures of eating disorder symptomology (the Eating Attitudes Test-26: Garner et al., 1982), but also implicit bias toward thinness.

Testing implicit attitudes has been hypothesized to allow researchers to access stimulus associations which participants may not have conscious access to, or may consciously attempt to over-ride when reporting on explicit measures (see, e.g., Wilson et al., 2000; Gawronski and LeBel, 2008). In the current study, we used a reaction time task based on the Implicit Association Test (IAT) (Greenwald et al., 1998), to test the relative automaticity of participants’ ability to pair slimmer bodies with more positively valenced words and vice versa.

Previous studies looking at how dissonance may change implicit attitudes have not generally found support for such an effect. For instance, Gawronski and Strack (2004) found that essay-based dissonance tasks on both alcohol and positive discrimination were able to shift explicit attitudes but did not change implicit attitude scores to these issues. Indeed, Gawronski and colleagues (Gawronski and Strack, 2004; Hofmann et al., 2005; Gawronski and Bodenhausen, 2006; Gawronski et al., 2007; Gawronski and LeBel, 2008) have argued that explicit attitudes are inherently representational while implicit attitudes are distinct in terms of being automated and associative, and thus dissonance should act at the representational level and not the associative. Related to this, interventions that have changed implicit attitudes have often used associative techniques to do so. For instance, on a conceptually related topic to ours, implicit self-esteem has been changed through training of associations (e.g., Grumm et al., 2009) and even by using the IAT as a means to help participants practice positive-self associations (Ebert et al., 2009).

Critically, although Succeed puts cognitive dissonance at the center of its approach to improving body image, it also differs considerably from standard experimental approaches to studying dissonance. Participants engage in not just one dissonance activity, but multiple exercises in varying forms (e.g., letter writing, role playing, reporting on activities to the group), spread across two 2-h sessions and homework in between. Furthermore, some of the exercises explicitly involve practicing positive body associations (e.g., practicing positive affirmations and self-complements out loud; see Supplementary Materials for more detail). As such the material incorporates elements that could be considered as practicing evaluative practice (both positive about the self, and negative regarding the thin ideal) and thus may be considerably more likely to induce changes at the implicit level than standard experimental interventions such as induced compliance in an essay writing task.

The primary aim of the current study was therefore to consider whether the Succeed program could impact on implicit attitudes to thinness. Assessing efficacy *via* implicit and explicit outcomes may deliver a holistic understanding of how they independently and synergistically predict thin-ideal adherence and disordered eating. Therefore, this study examined implicit changes, alongside explicit changes, *via* an Implicit Association Test-based task (IAT) and the Eating Attitudes Test-26 (EAT-26; Garner et al., 1982) respectively.

### Sexual Orientation and Body Dissatisfaction

A secondary aim of the current study was to consider sexual orientation as a potential moderator of program outcomes. The literature shows mixed findings regarding whether sexual minority women have higher risk (e.g., Austin et al., 2013; Matthews-Ewald et al., 2014) or lower risk (e.g., Owens et al., 2002; Diemer et al., 2015) of eating disordered behaviors. Some studies find that bisexual women are more at risk than lesbians (e.g., Austin et al., 2009; Polimeni et al., 2009), while other data suggests the risk among lesbians may be increasing over time (Watson et al., 2017). Studies focusing specifically on body image are likewise ambiguous. Some studies comparing lesbians with heterosexual women have tended to find that lesbians experience less body dissatisfaction and/or less weight preoccupation, and have higher body weight ideals, than heterosexual women (e.g., Strong et al., 2000; Morrison et al., 2004; Peplau et al., 2009). However, other recent studies also including bisexual women failed to find any group differences in body dissatisfaction (e.g., Davids and Green, 2011; Antfolk et al., 2017). This picture is further complicated by the fact that the drivers of body dissatisfaction and eating pathology may also differ between heterosexual individuals and those in sexual minorities (Davids and Green, 2011; Alvy, 2013; Huxley et al., 2015; Watson et al., 2015). As such, considering the potential for body image programs such as Succeed to benefit non-heterosexual women is important. Because the program materials as originally written are often heteronormative, we adjusted terminology to make the materials applicable to non-straight women; for instance one roleplay involving a young woman wanting to be thin for her boyfriend was removed, and other references to partners were made gender neutral (see Brown and Keel, 2015, and Jankowski et al., 2017, for related discussion of adapting these interventions for gay men).

We therefore recruited participants through LGBTa+ societies as well as more typical routes, in order to consider whether sexual orientation predicted improvements in eating attitudes and IAT scores across the experiment.

## Methods

### Participants

One hundred and twenty-five women were initially recruited. A mostly heterosexual sample was recruited through participant pools and word of mouth, while recruitment of non-heterosexual women was primarily through the LGBTa+ Societies of three British universities in North East England. Our recruitment strategy was to recruit as many participants as possible within the time-frame available to the two student authors (RK, AWC). All participants were ascribed female at birth and had a female gender identity. Following exclusions for elevated eating disorder symptoms and immediate withdrawals, 102 women aged 18–30 years (mean 19.4) took part in the study, of whom 93 provided complete questionnaire data at both time points (9 participants completed pre-test but not post-test), and 70 provided complete IAT data at both time points (more data were lost for the IAT due to the difficulties some participants had completing the test on their personal computers or devices). Participants who failed to complete post-test measures did not differ from other participants on pre-test EAT-26 scores, but did show higher pro-thin bias on the IAT (see Supplementary Materials).

### Ethics

The study was approved by the Durham University Psychology Ethics Committee. Following committee stipulations, study advertisements asked those with a current diagnosis of an eating disorder not to participate, and 13 individuals with a baseline score above 25 on the EAT-26 were excluded from the study. All participants were supplied with details of the university counseling services and eating disorder information sources on completion of the study.

### Experimental Groups

Participants reported their sexual orientation on a 7-point Kinsey scale (Kinsey et al., 1948) from 1 (exclusively opposite sex preference) to 7 (exclusively same sex preference). This allows more variation in sexual orientation than a simple “heterosexual,” “bisexual,” and “homosexual” identity categorization. Unfortunately, while there was strong representation of women scoring 1 and 2 (exclusively or almost exclusively heterosexual) in control and intervention conditions, only six women scoring 3 or more provided control group data (some of which was incomplete). We therefore divided our participants into three groups: the control group (predominantly heterosexual; Kinsey scores below 3, final *n* = 38); a predominantly heterosexual intervention group (Kinsey scores below 3, final *n* = 18); and a non-heterosexual intervention group (Kinsey scores all above 4; no women scoring 3 took part in the intervention, final *n* = 31).

### Outcome Measures

#### Eating Attitudes Test

The 26-item self-reported EAT-26 (Garner et al., 1982) is a Likert-scale dimensional measure of eating disorder symptoms, which asks participants to report the frequency of behaviors and thoughts such as avoidance of fat-rich foods, monitoring of calories consumed, feeling pressure or concern about their weight, and active bulimic behaviors. Although the measure can be broken into three sub-scales (*Dieting, Bulimia & Food Pre-occupation* and *Oral Control*), the total scores are also informative and were used here. Cronbach’s alpha for internal consistency in the current sample was good (*α =* 0.79).

#### Implicit Association Test

**Figure 1:**
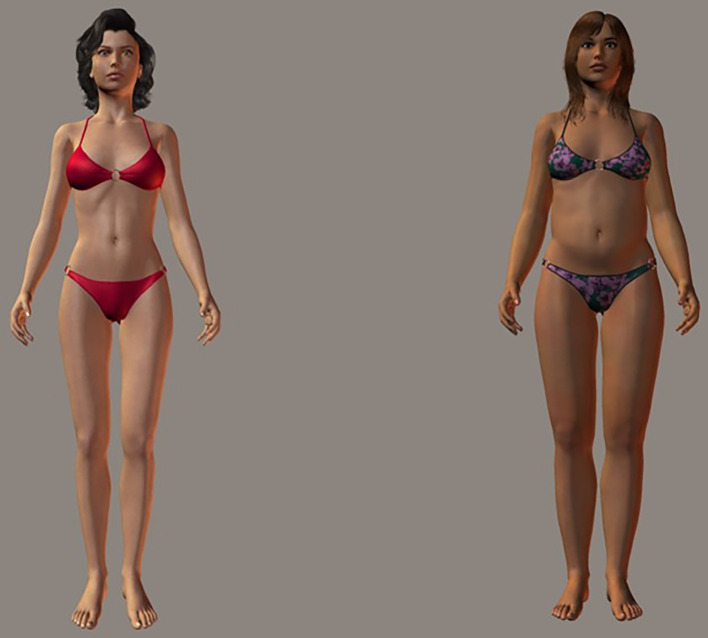
Example stimuli showing the slimmer and larger bodies used in the IAT.

Participants completed a two-choice reaction time task, based on the IAT. In a block of 16 trials, participants pressed the C-left or M-right key to indicate whether the stimulus fitted the categories on the left or right of the screen; these categories were either good/fat vs. bad/thin or good/thin vs. bad/fat. Stimuli were either words (4 “good” – beautiful, desirable, happy, successful, 4 “bad” – ugly, repellent, miserable, failure, each presented twice) or computer-generated images of women with putative BMIs of 17.2 and 30.9 (see Boothroyd et al., 2012, for more detail on how stimuli were constructed; example figures are shown in Figure 1). Participants completed 16 trials of words, then 16 trials of images, followed by 32 trials of words and images randomly interspersed, for a given category combination. They then completed the same process with the opposite combination. Once a stimulus was categorized correctly the stimulus was removed and the next stimulus was presented after a 400-ms delay. Incorrect categorization was indicated with an X, which remained on screen until the correct key was pressed. Participants were urged to complete the test quickly with minimal mistakes. Order of category pairings (thin + good versus thin + bad) and within-block stimuli were randomized to counterbalance participant practice effects. Implicit thin-ideal IAT D scores, which indicate the relative bias in reaction times to one combination of pairings vs. the other, controlling for participants’ general reaction time distribution, were calculated as per Greenwald et al. (1998).

### Procedure

Participants completed the consent form and the EAT-26 and IAT online at the point of recruitment and were allocated to complete either (1) the experimental Succeed Body Image Programme (SBIP) (final *n* = 56 with at least complete EAT-26 data) or (2) read NHS leaflets (final *n* = 37).

The Becker and Stice (2011) SBIP intervention was conducted across two 2-hour sessions, led by Succeed-trained peers, held a week apart in groups of 6–10 participants. The SBIP presented participants with information about the dangers of pursuing the thin ideal and the benefits of a healthy body ideal. Participants completed activities such as roleplays in which a “friend” had to be persuaded to reject the thin ideal, mirror tasks to notice one’s positive attributes, practice of affirmations, or strategies to avoid future body concerns and an empathetic letter to a struggling teenage girl explaining the biological, social, or financial damage of pursuing the thin-ideal. Further details of the activities are given in the Supplementary Materials.

Control group participants read National Health Service (NHS) leaflets titled (1) Eating Disorders (see most recent online version here: https://www.nhs.uk/conditions/eating-disorders/) and (2) Eating a Balanced Diet (see most recent online version here: https://www.nhs.uk/live-well/eat-well/), which included mental and physical health information similar to the SBIP but without the dissonance or practice elements. The Eating Disorders leaflet elaborated on the causes, symptoms, and treatments of varying eating disorders whereas the Balanced Diet contained material on the Eatwell plate and appropriate balance of food types.

A week after concluding the second session of the SBIP (or a similar time frame from baseline for those reading the NHS leaflets), participants completed the EAT-26 and IAT again. There was typically a 4-week interval between the baseline and post-test measures.

## Results

Table 1 shows correlations between variables for the full sample and for those with complete data on the EAT-26 or IAT. IAT and EAT-26 scores were not associated at baseline or post-test.

**Table 1:** Correlations between eating attitudes, sexual orientation, and IAT D scores for all eligible participants (above the diagonal) and those with complete IAT or EAT-26 data (below the diagonal).

		EAT-26 pre-test	EAT-26 post-test	IAT pre-test	IAT post-test
**EAT-26pre-test**	*r*		0.554^**^	0.057	0.196
	*p*		<0.000	0.598	0.121
	*n*		87	89	64
**EAT-26post-test**	*r*	0.554^**^		0.059	0.074
	*p*	<0.000		0.602	0.570
	*n*	63		80	61
**IATpre-test**	*r*	0.177	0.115		0.161
	*p*	0.169	0.372		0.212
	*n*	62	62		62
**IATpost-test**	*r*	0.165	0.081	0.134	
	*p*	0.207	0.539	0.312	
	*n*	60	60	59	

** *Correlation is significant at the 0.001 level (2-tailed)*.

**Figure 2:**
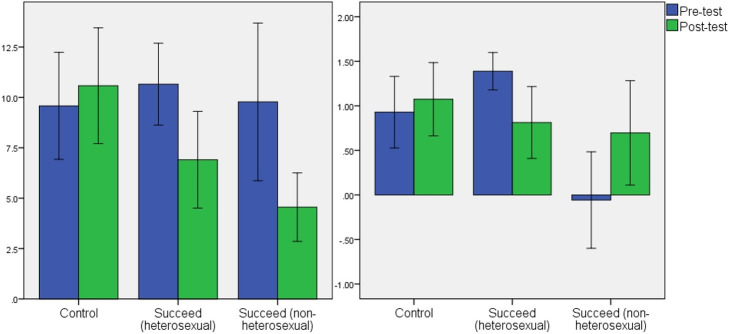
Mean scores with 95% confidence intervals for the association between time (pre-test and post-test) and group on EAT-26 scores (left panel) and IAT scores (right panel).

To test the effect of the SBIP intervention on eating attitudes and implicit thin-bias, repeated measures ANOVAs were run in SPSS 22, where time (pre-test or post-test) was the within-subjects variable and group (Succeed (predominantly heterosexual), Succeed (non-heterosexual), or control) was a between-subjects measure. Full results are shown in Table 2. For EAT-26 scores, the model showed no main effect of group. There was, however, a significant group by time interaction. Paired analyses within group showed that participants in both intervention groups experienced a significant decrease in their levels of disordered eating attitudes (predominantly heterosexual: *t* = 3.917, df = 37, *p* < 0.001; non-heterosexual *t* = 3.480, df = 17, *p* = 0.003) but the control group showed no such change (*t* = 0.992, df = 36, *p* = 0.328). Data are illustrated in Figure 2, and all means are given in Supplementary Materials.

**Table 2:** Results of ANCOVA models for effects of intervention on EAT-26 and IAT scores.

	df	*F*	*p*	Partial eta squared
**EAT-26**				
Group	2.84	1.278	0.284	0.030
Time	1.84	14.438	0.000	0.147
** Group × time**	**2.84**	**7.400**	**0.001**	**0.150**
**IAT**				
Group	2.59	84.371	0.000	0.588
Time	1.59	0.514	0.476	0.009
** Group × time**	**2.59**	**6.568**	**0.003**	**0.182**

*The key test of the experimental effect is the group by time interaction, shown in bold*.

There was a significant main effect of group for IAT score. As seen in Figure 2, women in the non-heterosexual group had overall IAT scores lower than the predominantly heterosexual groups (Tukey’s *post hoc* comparisons significant with *p* < 0.02). In fact, the mean at baseline was close to zero, indicating no pro- or anti-thin bias. There was also a significant interaction between time and group. Paired *t*-tests showed a significant drop in thin-bias in the predominantly heterosexual Succeed intervention group (*t* = 2.812, df = 24, *p* = 0.010). There was no change in the control group (*t* = 0.609, df = 20, *p* = 0.550). The non-heterosexual Succeed group showed a non-significant trend toward stronger pro-thin bias at post-test (*t* = 2.103, df = 15, *p* = 0.053).

## Discussion

The current study investigated the effect of the Succeed Body Image Programme, a cognitive-dissonance eating disorder intervention, on explicit and implicit measures of eating attitudes and thin-bias respectively, in a sexually diverse group of female undergraduates. Results showed a significant effect of the intervention on participants’ explicit self-reports of pathological eating attitudes, in both the predominantly heterosexual, and the non-heterosexual groups. On the other hand, implicit thin bias was significantly reduced by the intervention only among the predominantly heterosexual group. Although all “predominantly heterosexual” participants showed longer response times to the counter-cultural thin-bad/fat-good trials than the concordant thin-good/fat-bad trials, this effect became weaker between baseline and post-test in those who had completed the intervention, while those who had read NHS leaflets showed no change. Surprisingly, although the non-heterosexual group started off showing no pro- or anti-thin bias, following the intervention they did show a pro-thin bias (although we note the paired *t*-test for this group did not quite reach significance).

Our results for EAT-26 questionnaire data are consistent with recent systematic reviews of dissonance intervention trials which similarly produce reductions in eating disorder risk factors, such as thin-ideal internalization, body dissatisfaction, and self-reported dieting in intervention participants (see Pennesi and Wade, 2016, for review of all studies). Demonstrating an effect of this intervention on implicit attitudes among predominantly heterosexual women, *via* response latencies, is novel evidence that extensive dissonance interventions may indeed change underlying associative cognitions as well as explicit, self-reported beliefs among these participants. Because implicit measures are ostensibly not subject to response bias (Gawronski et al., 2007), it is unlikely that our participants were merely suppressing their thin-ideal associations in the intervention group. Rather it seems more likely that the repeated practice of these body-positive associations enabled genuine implicit change. Furthermore, our responses are consistent with those of Stice et al. (2015) who showed that completion of a similar dissonance-based program reduced neurological reward responses to thin-ideal imagery. Both their data and ours point toward the interventions reducing the overall positive associations with thinness in participants.

The SBIP meets the “gold standard” for eating disorder prevention *via* body image resilience, with effectiveness appearing to rest on it being interactive, multi-sessional, and dissonance-based instead of being didactic, single session, and psychoeducational (Stice and Shaw, 2004; Stice et al., 2019). As discussed above, however, traditional dissonance interventions have had difficulty in inducing implicit changes in participants. It seems likely therefore, that the repeated practice of positive associations and anti-thin-ideal associations may contribute to the implicit changes observed here in the predominantly heterosexual intervention group, beyond the simple inclusion of dissonance. Given that one arm of the program is to equip women with affirmations that they can repeat to themselves regularly and at key junctures in the future, it is possible that testing implicit attitudes further from the point of intervention may show stronger effects.

It remains puzzling, however, why non-heterosexual women should appear to benefit from the Succeed intervention in terms of their self-reported eating attitudes, but show a trend toward an inverse relationship for their implicit attitudes. One possibility is that their very lack of such implicit bias at baseline meant that discussion of the thin ideal during the sessions actually strengthened the salience of this stereotype and thus rendered them more prone to a bias in response latencies. As such, although we can conclude from the current study that the intervention seemed to work as intended among this diverse sample of women as far as explicit eating attitudes are concerned, we cannot draw clear conclusions regarding what underlying mechanisms (such as change in underlying associations) *cause* those changes in eating attitudes. Indeed, is it entirely possible that different groups of women may experience benefits from the program in different ways, just as women of different sexual orientations may experience eating disorder risks in different ways (Davids and Green, 2011; Alvy, 2013; Huxley et al., 2015; Watson et al., 2015).

We note a particular caveat relating to this non-straight sample in our study, however. Our overall sample size was hampered by difficulties some participants experienced with the IAT task, and we had poorer completion in the control condition. As regards non-heterosexual participants, we were unable to further subdivide our participants into sexual orientation groups as there was insufficient representation of participants across the Kinsey scale to effectively split into bisexual and lesbian. Furthermore, as sexual orientation scores were unevenly spread between the control and intervention conditions, we were unable to form any comparable non-heterosexual control group. This is a clear limitation and future research would benefit from recruiting participants in larger numbers (we were largely limited by recruitment through just three LGBTa+ societies).

We also did not address the broader range of sexualities (e.g., a- and demi-sexual, and “pansexual,” “heteroflexible,” “queer”; Callis, 2014). Given that these groups may all differ in key respects (e.g., Chmielewski and Yost, 2013; Smalley et al., 2016) and some studies have found that some sexual orientation subgroups may be more at risk than others (e.g., “pan”: Himmelstein et al., 2019; or “unsure”: Diemer et al., 2015), further research should consider the impact of sexual orientation on eating disorder interventions in more detail. We also note that we have concentrated on sexual orientation in women but that sexual orientation is also important in body image and eating pathology in men (as discussed above, a similar intervention has been adapted for gay men: see Brown and Keel, 2015; Jankowski et al., 2017) and that gender identity may also be an important factor to consider. Trans individuals may have particularly high risk of pathological eating attitudes and/or unhealthy weight control behaviors (e.g., Diemer et al., 2015; Brewster et al., 2019) and may particularly benefit from targeted intervention work.

We further note that we used only the EAT-26 as our explicit measure of attitude changes over time, and not other measures such as those explicitly addressing body esteem (both positive and negative) and thin-ideal internalization. Our implicit measure likewise only addressed whether participants associated slimmer figures with positive social attributes, and did not address their own self-concept or associations with food. We would therefore suggest further research not only replicating the current results but also broadening the scope of which aspects of implicit cognitions relating to body esteem and eating disorders are considered. Other forms of implicit attitudes may also be worthy of investigation, such as the Implicit Relational Assessment Procedure (IRAP) which is similar to the IAT; yet it adds relational components to assess directionality and relations between concepts, and was used by Timko et al. (2010) to assess implicit body image dissatisfaction.

In conclusion, we have demonstrated that an immersive, intensive dissonance-based body image intervention may be able to impact implicit attitudes *via* associative response latencies among predominantly heterosexual women, while non-heterosexual women experienced benefits in explicit eating attitudes but no significant change in thin-bias. Further research, however, is badly needed to confirm these results and further establish (1) the extent to which implicit attitude change may be seen across related associations and over time and (2) how sexual orientation or identity moderates the effects in women.

## Data Availability Statement

All datasets generated for this study are included in the article/Supplementary Material.

## Ethics Statement

The studies involving human participants were reviewed and approved by Durham University Psychology Department Ethics Committee. The participants provided their written informed consent to participate in this study.

## Author Contributions

All authors contributed to study design. ES programmed test materials. RK and AW-C collected the data. LB performed data analyses. RK and LB wrote the manuscript with input from all authors.

### Conflict of Interest

The authors declare that the research was conducted in the absence of any commercial or financial relationships that could be construed as a potential conflict of interest.

## Supplementary Material

DATA SHEET S1 Final data set.

DATA SHEET S2 Supplementary material and code.
