# Corrigendum: The Impact of a Dissonance-Based Eating Disorders Intervention on Implicit Attitudes to Thinness in Women of Diverse Sexual Orientations

**DOI:** 10.3389/fpsyg.2020.604557

**Published:** 2020-10-22

**Authors:** R. M. Naina Kant, Agnes Wong-Chung, Elizabeth H. Evans, Elaine C. Stanton, Lynda G. Boothroyd

**Affiliations:** ^1^Department of Psychology, Durham University, Durham, United Kingdom; ^2^School of Psychology, Newcastle University, Newcastle upon Tyne, United Kingdom

**Keywords:** cognitive dissonance, body image, intervention, eating disorders, implicit attitudes

In the published article, there was an error in the Supplementary Data Sheet S1 as published. The original supplementary data included information which created a small risk of participant re-identification. This data has now been replaced with a fully anonymized file.

The authors apologize for this error and state that this does not change the analyzed variables or scientific conclusions of the article in any way. The original article has been updated.

