# Supplementary material for: The Impact of a Dissonance-Based Eating Disorders Intervention on Implicit Attitudes to Thinness in Women of Diverse Sexual Orientations
Source: Front Psychol. 2019 Nov 29;10:2611. doi: 10.3389/fpsyg.2019.02611 (PMC6895132; doi:10.3389/fpsyg.2019.02611)
Supplement: DATA SHEET S2 — Supplementary material and code. [file Data_Sheet_2.PDF]

**The impact of a dissonance-based eating disorders intervention on implicit attitudes to thinness in women of diverse sexual orientations.**

Kant, Wong-Chung, Evans & Boothroyd

**Supplementary Material**

## 1. Participant non-completion

Means and standard deviations for participants with and without complete data are shown in Table S1 below. There was no significant difference on Mann Whitney tests of initial EAT-26 scores for participants who did or did not complete the study ($z$ = -.384, $p$ = .701) although participants who did not complete had more pro-thin bias on the IAT ($z$ = -2.273, $p$ = 023). Whether or not participants were able to provide complete IAT data did not relate to their initial scores ($z$ = -1.176, $p$ = .240; IAT: $z$ = -.277, $p$ = .782).

Table S1. Descriptive statistics for participants with and without complete data on each measure.

| | | Age | EAT-26 pre-test | EAT-26 post-test | IAT pre-test | IAT post-test |
|---|---|---|---|---|---|---|
| Incomplete EAT data | Mean | 19.88 | 11.44 | | 1.5667 | 1.5187 |
| | N | 8 | 9 | | 9 | 3 |
| | SD | 1.126 | 8.033 | | .31287 | .41919 |
| Complete EAT data | Mean | 19.60 | 10.09 | 7.73 | .8186 | .8415 |
| | N | 82 | 87 | 87 | 80 | 61 |
| | SD | 1.735 | 6.867 | 7.212 | .97432 | .98822 |
| Incomplete IAT data | Mean | 19.93 | 8.87 | 7.58 | .9495 | 1.7803 |
| | N | 27 | 30 | 24 | 24 | 1 |
| | SD | 1.517 | 6.146 | 7.136 | .95672 | . |
| Complete IAT data | Mean | 19.49 | 10.83 | 7.79 | .8739 | .8589 |
| | N | 63 | 66 | 63 | 65 | 63 |
| | SD | 1.749 | 7.244 | 7.297 | .96139 | .97898 |

## 2. Intervention contents

| | |
|---|---|
| Session 1 | |
| Defining the thin ideal and its origins | Participants extensively brainstorm the components of 'the perfect woman' in their culture. The resulting list is ret=-titled 'Thin Ideal' and is actively contrasted through discussion of historical and capitalist issues with the 'Healthy Ideal'. |
| Costs of the thin ideal | Participants produce a list of costs associated with the thin ideal under the categories: self-esteem, health, academic, quality of life, and costs to society and their community (e.g. university or college). Participants are challenged to consider whether these costs are worthwhile. |
| Verbal challenges | Participants describe up to three scenarios in which they felt pressure to be thin. They report to the group what they said at the time, and what they would say now in order to actively resist that pressure. Peer leaders encourage participants to give these responses with confidence and practice if needed. |
| Challenging fat talk | The group identify statements used in body-related conversation and discuss how they maintain the thin ideal. |
| Homework | |
| Letter to a teenage girl | Participants write a letter to a young girl struggling with body image, and explain the costs of the thin ideal in order to encourage her to reject it. |
| Mirror exercise | Participants stand in front of a mirror and record as many positive qualities about themselves as they can. They are asked to produce at least 15, including physical, social and emotional characteristics. |
| Session 3 | |
| Homework debrief | Every participant reads their letter out loud and reflect on how they felt writing it. Participants discuss how the mirror exercise felt and what was most challenging about it. |
| Role play discussions | Participants split into groups; for each group a peer leader plays the part of a young woman committed to the thin ideal within a given scenario. The participants must prepare and then deliver arguments to convince her that the thin-ideal is costly and not worth pursuing. The peer leader resists their arguments and forces them to exhaustively persuade her. |
| Body activism | Participants brainstorm actions they can take (or avoid) to battle the thin ideal on a personal and social level. They also consider possible barriers and how these can be overcome. |
| Future pressures | Participants imagine future scenarios in which they may feel under appearance pressure (e.g. pregnancy, weddings, middle age changes) and consider how to resist that pressure. |
| Self-affirmation | Participants verbally commit to challenging appearance pressures themselves, by specific actions going forward (e.g. repeating the mirror exercise, actively challenging fat talk). |

## 3. Supplementary results

Table S2. Descriptive statistics for participants overall and by experimental group.

| Group | | Age | EAT-26 pre-test | EAT-26 post-test | IAT pre-test | IAT post-test |
|---|---|---|---|---|---|---|
| Control group | Mean | 19.32 | 9.74 | 10.58 | .8692 | 1.0745 |
| | N | 34 | 35 | 31 | 33 | 21 |
| | SD | 1.093 | 7.298 | 7.831 | .88565 | .90313 |
| Intervention group (straight) | Mean | 19.34 | 10.79 | 6.91 | 1.2959 | .8217 |
| | N | 38 | 43 | 38 | 40 | 27 |
| | SD | 1.097 | 6.360 | 7.308 | .69288 | .96864 |
| Intervention group (non-striaght) | Mean | 20.78 | 9.78 | 4.56 | -.0580 | .6961 |
| | N | 18 | 18 | 18 | 16 | 16 |
| | SD | 2.861 | 7.863 | 3.417 | 1.01659 | 1.09924 |
| Total | Mean | 19.62 | 10.22 | 7.73 | .8943 | .8733 |
| | N | 90 | 96 | 87 | 89 | 64 |
| | SD | 1.687 | 6.948 | 7.212 | .95528 | .97799 |

## 4. SPSS Syntax for all analyses

```
COMPUTE filter_$=(Eligible = 1 & Group_3cat >  -1).
VARIABLE LABELS filter_$ 'Eligible = 1 & Group_3cat >  -1 (FILTER)'.
VALUE LABELS filter_$ 0 'Not Selected' 1 'Selected'.
FORMATS filter_$ (f1.0).
FILTER BY filter_$.
EXECUTE.

GLM IAT_D_Time1 IAT_D_Time2 BY Group_3cat
  /WSFACTOR=time 2 Polynomial
  /METHOD=SSTYPE(3)
  /EMMEANS=TABLES(Group_3cat*time)
  /PRINT=ETASQ PARAMETER
  /CRITERIA=ALPHA(.05)
  /WSDESIGN=time
  /DESIGN=Group_3cat.

GLM EAT26_time1 EAT26_time2 BY Group_3cat
  /WSFACTOR=time 2 Polynomial
  /METHOD=SSTYPE(3)
  /EMMEANS=TABLES(Group_3cat*time)
  /PRINT=ETASQ PARAMETER
  /CRITERIA=ALPHA(.05)
  /WSDESIGN=time
  /DESIGN=Group_3cat.

GRAPH
  /BAR(GROUPED)=MEAN(IAT_D_Time1) MEAN(IAT_D_Time2) BY Group_3cat
  /MISSING=LISTWISE
  /INTERVAL CI(95.0).
```

```
GRAPH
  /BAR(GROUPED)=MEAN(EAT26_time1) MEAN(EAT26_time2) BY Group_3cat
  /MISSING=LISTWISE
  /INTERVAL CI(95.0).

SORT CASES  BY Group_3cat.
SPLIT FILE LAYERED BY Group_3cat.
T-TEST PAIRS=IAT_D_Time1 EAT26_time1 WITH IAT_D_Time2 EAT26_time2
(PAIRED)
  /CRITERIA=CI(.9500)
  /MISSING=ANALYSIS.
SPLIT FILE OFF.

#Additional analyses for supplementary material

MEANS TABLES=Age EAT26_time1 EAT26_time2 IAT_D_Time1 IAT_D_Time2 BY
complete_EAT complete_IAT
  /CELLS=MEAN COUNT STDDEV.

MEANS TABLES=Age EAT26_time1 EAT26_time2 IAT_D_Time1 IAT_D_Time2 BY
Group_3cat
  /CELLS=MEAN COUNT STDDEV.

NPAR TESTS
  /M-W= EAT26_time1 EAT26_time2 IAT_D_Time1 IAT_D_Time2 BY
complete_EAT(0 1)
  /M-W= EAT26_time1 EAT26_time2 IAT_D_Time1 IAT_D_Time2 BY
complete_IAT(0 1)
  /MISSING ANALYSIS.


CORRELATIONS
  /VARIABLES=EAT26_time1 EAT26_time2 IAT_D_Time1 IAT_D_Time2
  /PRINT=TWOTAIL NOSIG
  /MISSING=PAIRWISE.
SORT CASES  BY Complete.
SPLIT FILE LAYERED BY Complete.
CORRELATIONS
  /VARIABLES=EAT26_time1 EAT26_time2 IAT_D_Time1 IAT_D_Time2
  /PRINT=TWOTAIL NOSIG
  /MISSING=PAIRWISE.
SPLIT FILE OFF.
```
